# Clinical Significance of PEAK1 Expression and BRAF V600E Mutation in Papillary Thyroid Cancer

**DOI:** 10.3389/bjbs.2021.10268

**Published:** 2022-01-11

**Authors:** P. Li, H. Zhao, X. Liu, Y. Huang, D. Chen

**Affiliations:** ^1^ Department of Endocrinology, The Affiliated Hospital of Qingdao University, Qingdao, China; ^2^ Department of Pathology, The Affiliated Hospital of Qingdao University, Qingdao, China; ^3^ Department of Nuclear Medicine, The Affiliated Hospital of Qingdao University, Qingdao, China; ^4^ Department of Otorhinolaryngology, The Affiliated Hospital of Qingdao University, Qingdao, China; ^5^ Department of General Surgery, The Affiliated Hospital of Qingdao University, Qingdao, China

**Keywords:** Hashimoto’s thyroiditis, BRAF V600E mutation, PEAK1, radioactive iodine response, papillary thyroid cancer

Papillary thyroid cancer (PTC) is a common endocrine malignancy, which accounts for 80–85% of all thyroid cancers. Overall, the prognosis of PTC is very good. However, for those with tumor invasion, metastasis or refractory PTC, the prognosis of these patients is poor and eventually die from postoperative recurrence and metastasis (1). Therefore, predicting the prognosis of papillary thyroid carcinoma and customizing individualized treatment programs have become topics of concern for clinicians.

At present, surgical resection is the first choice for the treatment of PTC. After thyroidectomy, adjuvant treatment is usually given according to the severity of the disease and the metastasis of the tumor. Iodine-131 (I^131^) treatment is the most common among various adjuvant treatments. Tumor cells derived from thyroid tissue usually retain the ability to take up iodine. I^131^ treatment can effectively eliminate residual thyroid tissue and micrometastasis. Therefore, this treatment plays an important role in reducing the recurrence rate and improving the survival rate. Predictive evaluation of the efficacy of I^131^ treatment and development of a corresponding degree of chemotherapy can effectively improve the prognosis of PTC patients and prolong the patient’s recurrence-free survival.

BRAF represents one of the most frequently mutated protein kinase genes in human tumors. The T1779A point mutation in BRAF exon 15, which results in a V600E amino acid substitution, is the most common mutation and accounts for more than 90% of all mutations found in the BRAF gene (2). Studies have demonstrated an association of BRAF V600E mutation with aggressive clinicopathologic characteristics of PTC, PTC recurrence and I-131 treatment response (3). However, several other studies have found that BRAF status was not associated with negative prognostic features and I-131 treatment response (4).

Pseudopodium enriched atypical kinase 1 (PEAK1or Sgk269) is a non-receptor tyrosine kinase which is enriched in the pseudopodia of migrating cells and played an important role in regulating cell migration, proliferation and chemosensitivity (5). High PEAK1 expression was significantly associated with advanced clinical stage and poor prognosis in many cancers. However, the expression and role of PEAK1 in human PTC is unknown. In the present study, we evaluate the PEAK1 expression and BRAF V600E mutation in patients with PTC who received total thyroidectomy following I^131^ therapy, hypothesising links between PEAK1 expression and BRAF V600E mutation with clinicopathologic characteristics and I^131^ therapy response.

We retrospectively screened 207 consecutive patients with PTC who received total thyroidectomy following I^131^ therapy between February 2015 and March 2016. All the patients were undergone postoperative TSH and I^131^ treatment was in accordance with the 2015 guidelines of the American Thyroid Association (ATA) (6). Patients were classified using the AJCC/TNM 8th Edition. Response to initial therapy was assessed by the ATA and classified as follows: 1) excellent response; 2) indeterminate response; 3) biochemically incomplete response or 4) structurally incomplete response (6). The Institutional Review Board of the affiliated hospital of Qingdao University approved the study. Written informed consent was obtained from all patients.

Two sets of TMA (Tissue microarray) were constructed for this study from paraffin-embedded tissue blocks. TMA set 1 consists of tissue cores taken from a cohort of 207 patients with PTC. TMA set 2 included tissue cores from the adjacent normal tissues. Areas of interest were marked by a pathologist and 0.6 or 1 mm diameter of cores were taken from the blocks and TMA were constructed. Several representative 1–1.5 mm cores were taken from diagnostic biopsies to construct the TMA. Four micrometer sections were placed onto Superfrost Plus poly-L-lysine coated glass slides (Thermo Fisher Scientific, IL, United States), and baked overnight at 37°C in a tissue drying oven.

Each specimens was mounted on adhesive-coated slides, deparaffinized, and rehydrated through xylene and alcohol. For antigen retrieval, slides were heated in the microwave for 10 min in citrate buffer (pH 6.0), followed by a 15 min cooling period. Endogenous peroxidase was quenched with aqueous 3% H_2_O_2_ for 15 min and washed with 1xPBS using 0.5% Tween 20 solution. The slides were blocked with 3% BSA in PBS for 30 min and subsequently incubated with PEAK1 antibody (1:200; Sigma-Aldrich, Beijing, China) for 1 h, followed by the biotinylated goat anti-mouse IgG (1:400; promocell, Shanghai, China) for 30 min, then by the Elite ABC Kit (STANDARD, Shanghai, China) for 30 min, and the DAB chromagen for 5 min, which resulted in a brown-colored precipitate at the antigen site. The slides were counterstained with hematoxylin. Sections were dehydrated, cleared, and mounted using DPX.

Cytoplasmic immunoreactivity for PEAK1 was evaluated according to the extent of staining in the cells. For statistical analysis, we grouped patients into two groups, positive expression being defined by staining of more than 10% of the tumour cells. Tumors or normal tissues with less than 10% of the cells with nuclear or membrane staining were considered negative PEAK1 expression. Hematoxylin‐eosin slides prepared from specimens were examined to estimate the areas enriched in tumor cell populations. Tissue was scraped from this preselected area and transferred to an Eppendorf tube for DNA extraction using the QIAamp FFPE Tissue Kit (Qiagen). Using the forward primer 5′‐CTC​TTC​ATA​ATG​CTT​GCT​CTG​ATA​GG‐3′ and the reverse primer 5′‐AGT​TGA​GAC​CTT​CAA​TGA​CTT​TCT​AGT‐3′, exon 15 of the BRAF gene, which potentially contained the T1799A transversion mutation (encoding BRAF V600E), was amplified by PCR. Amplification was performed under the following conditions: one cycle at 95°C for 5 min, 35 cycles of denaturation at 95°C for 30 s, annealing at 55°C for 30 s, and extension at 72°C for 40 s; followed by a final extension at 72°C for 5 min using Premix Taq™ Hot Start Version (Takara). The purified PCR products were sequenced using the forward primer above and a BigDye Terminator v 3.1 kit (Thermo Fisher). Capillary separation and data collection were performed on an ABI 3500 Genetic Analyzer.

Statistical analysis was performed using SPSS 22.0 soft. The results were expressed as either a percentage or median and Interquartile Range (IQR). The association between PEAK1 expression and BRAF^V600E^ mutation and other clinicopathological characteristics was evaluated using χ ^2^ test and Fisher’s exact test. The clinical response to RAI therapy between positive and negative PEAK1 expression or BRAF^V600E^ mutation was compared using Mann-Whitney U-test. Multinomial logistic regression was adopted for multivariate analysis. *p* < 0.05 was considered statistically significant.

Of the 207 PTC patients, 84.5% (175/207) patients were positive PEAK1 expression. The vast majority of PEAK1 was overexpressed in the cytoplasm (91.4% [160/175]) (Figures 1A,B), 8.6% (5/175) was expressed in the cell membrane and cytoplasm (Figure 1C), 15.5% (32/207) was negative PEAK1 expression in the PTC (Figure 1D) and no obvious nuclear expression was seen. The adjacent non-cancerous tissues were collected >2 cm from the tumor margins on the same lobe or on the opposite lobe. Of the adjacent normal tissues from 48 patients, three cases were subacute thyroiditis tissues, 10 cases were Hashimoto’s Thyroiditis, nine cases were nodular goiter and 26 cases were normal thyroid tissues. One patient (1/3) was positive PEAK1 expression in the subacute thyroiditis tissues (Figures 1E,F), five patients (5/10) were positive PEAK1 expression in the Hashimoto’s Thyroiditis (Figures 1G,H), four patients (4/9) were positive PEAK1 expression in the nodular goiter (Figures 1I,J) and 13 patients (13/26) were positive PEAK1 expression in the normal thyroid tissues (Figures 1K,L). PEAK1 was all expressed in the cytoplasm. The total positive PEAK1 expression rate in adjacent normal tissues was 47.9% (23/48), which was significantly lower than the tumor tissues (X^2^ = 4.381, *p* = 0.036).

**FIGURE 1:**
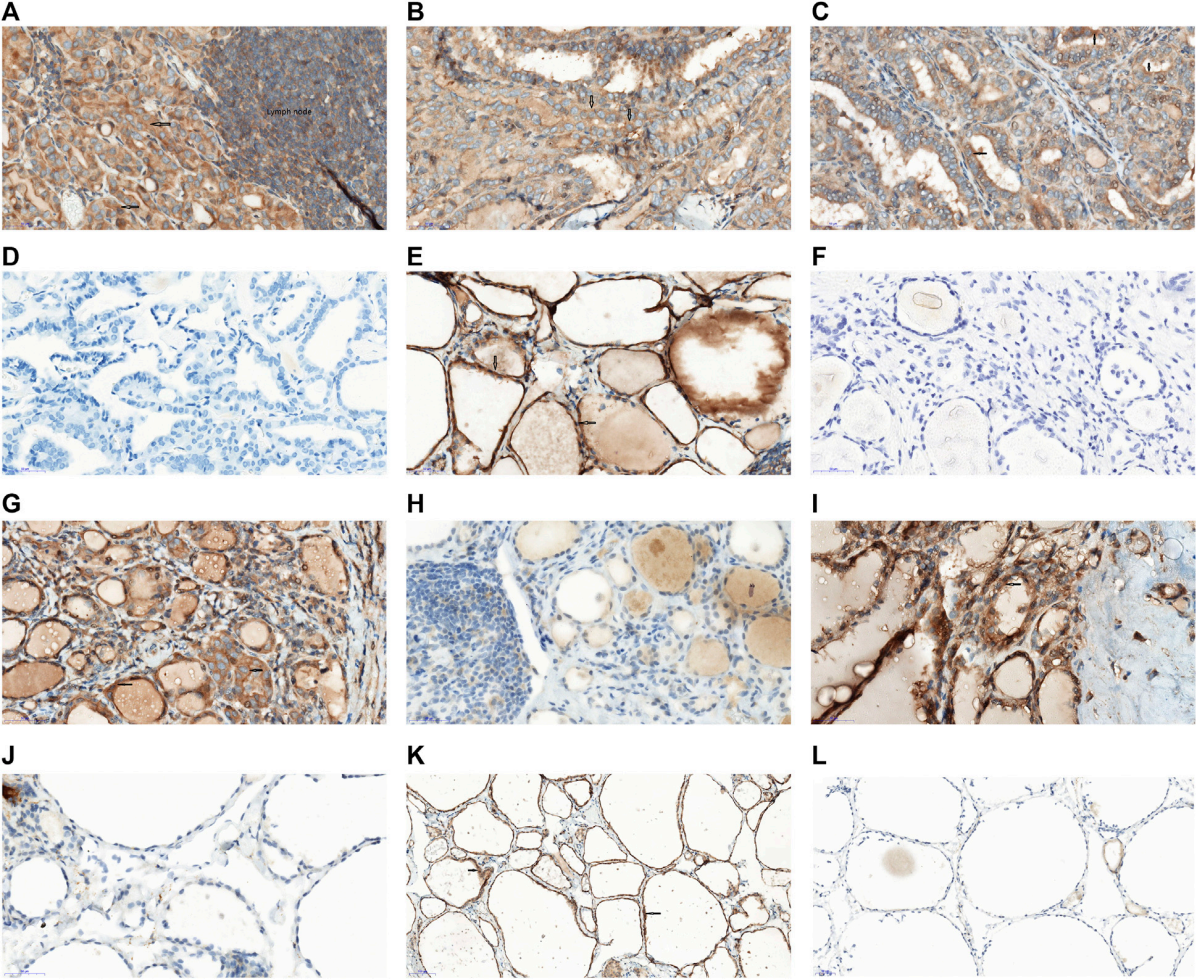
Expression of PEAK1 in PTC and adjacent normal tissues by IH staining. Immunohistochemistry of PEAK1 in the PTC tissues and adjacent normal tissues. Positive PEAK1 expression was shown in the cell membrane and cytoplasm, brown. The arrow represents the positive PEAK1 expression. **(A)** Representative positive PEAK1 expression in the cytoplasm of the PTC with the lymph node metastasis. **(B)** Representative positive PEAK1 expression in the cytoplasm of the PTC without lymph node metastasis. **(C)** Representative positive PEAK1 expression in the cell membrane and cytoplasm. **(D)** Representative negative PEAK1 expression in the PTC. **(E)** Representative positive PEAK1 expression in the cytoplasm of subacute thyroiditis tissues. **(F)** Representative negative PEAK1 expression in the cytoplasm of subacute thyroiditis tissues. **(G)** Representative positive PEAK1 expression in the cytoplasm of Hashimoto’s Thyroiditis. **(H)** Representative negative PEAK1 expression in the cytoplasm of Hashimoto’s Thyroiditis. **(I)** Representative positive PEAK1 expression in the cytoplasm of nodular goiter. **(J)** Representative negative PEAK1 expression in the cytoplasm of nodular goiter. **(K)** Representative positive PEAK1 expression in the cytoplasm of normal thyroid tissues. **(L)** Representative negative PEAK1 expression in the cytoplasm of normal thyroid tissues. (Magnify 400 times, ×400); Red arrow is PEAK1 membrane expression; Black arrow is PEAK1 cytoplasm expression.

Links between PEAK1 expression and the clinicopathological characteristics in PTC patients were determined (Table 1) Positive PEAK1 expression was associated with TNM stage, lymph node metastasis, extrathyroidal Invasion and high ATA risk, but not related with persistence/recurrence.

**TABLE 1:** Clinical and pathologic characteristics of PEAK1 expression and BRAF^V600E^ expression status in PTCs.

Groups	PEAK1 expression			BRAF^V600E^ expression		
	−(*n* = 32)	+(*n* = 175)	*p*-value	+ (*n* = 127)	−(*n* = 80)	*p*-value
Sex			0.743			0.576
Male	7	43		29	21	
Female	25	132		98	59	
Age (year)			0.442			0.116
<45	13	84		65	32	
≥45	19	91		62	48	
Tumor size			0.079			0.199
≥2 cm	12	40		28	24	
<2 cm	20	135		99	56	
Multifocality			0.909			0.129
Yes	13	73		58	28	
No	19	102		69	52	
TNM stage			0.025			0.112
I + II	18	132		97	53	
III + IV	14	43		30	27	
Lymph node metastasis			0.022			0.077
Yes	7	76		57	26	
No	25	99		70	54	
Extrathyroidal invasion			0.01			0.145
Yes	15	43		31	27	
No	17	132		96	53	
AJCC stage			0.133			0.185
I	25	154		113	66	
II + III + IV	7	21		14	14	
ATA risk stratification			0.005			0.227
Intermediate	22	154		111	65	
High	10	21		16	15	
Persistence/recurrence			0.169			0.044
Yes	5	14		22	6	
No	27	161		105	74	
PEAK1 expression						0.298
−	32			17	15	
+		175		110	65	

Of the 207 PTC patients, 61.4% (127/207) was the BRAFV600E mutation and 38.6% (80/207) was the BRAFV600E wild type by the retrospective study. BRAFV600E mutation was related with persistence/recurrence, but not related with other pathologic characteristics in the 207 PTCs (Table 1). No relationship was shown in PTC patients between PEAK1 expression and BRAFV600E mutation (Table 1).

Of the 127 PTC patients with BRAF^V600E^ mutation receiving radioiodine therapy, 51.9% (66/127) showed excellent response, 12.6% (16/127) showed indeterminate response and 35.4% (45/127) showed incomplete response. Of the 80 PTC patients without BRAF^V600E^ mutation receiving I^131^, 58.7% (47/80) showed excellent response, 8.8% (7/80) showed indeterminate response and 32.5% (26/80) showed incomplete response. BRAF mutation was not significant contributors to I^131^ response in PTC (*p* = 0.551). Of the 175 PTC patients with positive PEAK1 expression receiving I^131^, 60% (105/175) showed excellent response, 9.1% (16/175) showed indeterminate response and 30.8% (54/175) showed incomplete response. Of the 32 PTC patients with negative PEAK1 expression receiving I^131^, 25% (8/32) showed excellent response, 21.8% (7/32) showed indeterminate response and 53.1% (17/32) showed incomplete response. Patients with higher PEAK1 expression achieved better response, and lower PEAK1 expression achieved poor response (*p* = 0.001).

The BRAF V600E mutation has emerged as the promising molecular marker to predict prognosis in PTC. However, there are contradicting views on the role of BRAF mutation as a risk predictor in PTC. Xing et al. (7) reported that BRAF V600E mutation was significantly associated with larger tumor size, extrathyroidal invasion, lymph node metastasis, disease stage III/IV and strongly associated with persistent or recurrent disease. Yan et al. (8) reported that the presence of BRAF V600E mutation does not link to an aggressive prognosis or poor clinical outcomes in PTC. However, Walczyk et al. (4) reported no link between BRAF-positive primary focus of papillary microcarcinoma and more aggressive or recurrent disease. Therefore, there is still controversy as to whether the BRAFV600E mutation is a negative prognostic indicator. In the present study, BRAF V600E mutation was only related with recurrence in patients with PTC who received total thyroidectomy following I^131^ therapy, but not related with other aggressive prognoses.

The BRAF V600E mutation significantly reduces sodium-iodide symporter (NIS) expression and radioiodine uptake ability and influences I^131^ therapy to the point of causing I^131^-refractory PTC. Some studies reported that the BRAF mutation status may not impact the clinical response to I^131^ therapy for PTC patients, and the response to I^131^ therapy was not significantly different between the BRAFV600E mutation and the wild-type groups (9). However, Domínguez Ayala et al. (10) reported that the mutation of the BRAF V600E gene is related with greater resistance to postoperative treatment with I^131^ since the onset of the disease. In our study, BRAF mutation was not related with the response to I^131^ therapy.

PEAK1 is overexpressed in many human malignancies (11, 12). High PEAK1 expression is associated with a worse prognosis breast cancer and pancreatic cancer (11, 12). In our study, PEAK1 was overexpressed in PTC tissues. PEAK1 overexpression was related with higher TNM stage, lymph node metastasis, extrathyroidal Invasion and ATA risk stratification. These data indicated that PEAK1 overexpression is related to the invasive characteristics and represents the poor prognosis in PTC. Although PEAK1 overexpression was related with the aggressive characteristics of invasion and metastasis, PTC patients with higher PEAK1 expression has good response to I^131^ therapy. Therefore, detection of PEAK1 expression could predict the sensitivity to I^131^ therapy in PTC patients.

Our major contribution to the global literature is that patients with the BRAF V600E mutation are more likely to have persistence/recurrence than patients with wild-type BRAF. Therefore, PEAK1 expression and BRAF mutation may be a useful risk prognostic marker, and PEAK1 expression may predict radioactive iodine responses in patients with PTC. This work represents an advance in biomedical science because it shows that PEAK1 overexpression is related to good response to I^131^ treatment, invasion, and lymph node metastases.

## Data Availability

The original contributions presented in the study are included in the article/supplementary material, further inquiries can be directed to the corresponding author.
